# S11-1 From physical activity prescription to an active lifestyle: optimising the transfer

**DOI:** 10.1093/eurpub/ckad133.053

**Published:** 2023-09-11

**Authors:** Chair(s):, Alexis Lion, Eszter Füzéki, Suzanne McDonough, Klaus Pfeifer, Luc Lipkens, Susana Aznar, Sylvia Titze

**Affiliations:** Fédération Luxembourgeoise des Associations de Sport de Santé, Luxembourg; Goethe University, Frankfurt, Germany; Royal College of Surgeons in Ireland, Dublin, Ireland; Department of Sport Science and Sport, Friedrich-Alexander-Universität Erlangen-Nürnberg, Germany; Flanders Institute of Healthy Living NGO; PAFS Research group, Faculty of Sports Sciences, University of Castilla-La Mancha, Spain; University of Graz, Institute of Human Movement Science, Sport and Health, Graz, Austria

## Abstract

**Introduction:**

Physical activity (PA) is recommended to prevent and manage chronic diseases. Based on a wealth of scientific evidence, the World Health Organization (WHO) published specific PA recommendations for people with chronic diseases. However, people with chronic diseases are still not sufficiently physically active. To tackle this issue, the WHO published a recommendation to “implement systems of patient assessment and counselling on PA in primary and secondary health care and social services”. This symposium will present 4 European examples that (are willing to) implement strategies in healthcare settings to refer patients to PA programs.

**Project/policy description:**

In Germany, an ongoing research project (BewegtVersorgt) aimed to develop, implement, and evaluate a PA referral scheme (PARS) to promote PA for persons with chronic diseases within the German healthcare system. The PARS was coproduced by twelve health sectors organisations under the guidance of a research team.

In Flanders (Belgium), general practitioners (GPs) can use PA Prescription (PAP) since 2017. More recently, a collaboration between different European regions was set up to implement a European Physical Activity on Prescription model (EUPAP), based on the Swedish model. In this model, all healthcare professionals can use PAP. Barriers and facilitators of the implementation of EUPAP in Flanders have been identified.

In Spain, a national PAP plan was developed by the creation of exercise units that will work together with PA referral (PAR) in all autonomic Communities in Spain. Digital tools are used to improve the accessibility and efficacy of the Community Sport and Health systems to provide PA for the inactive population with or without chronic disease.

In Austria, the Jackpot.fit program was developed by Austrian health insurance funds and aims to refer physically inactive adults via several approaches: letter sent by the health insurance company, word of mouth, newspapers, sport-club trainers, and GPs. The letter sent by the health insurance company was much more efficient in terms of patient referral than the other approaches.

**Conclusions:**

These examples will open a discussion between the speakers and the symposium audience and will feed into the reflections of the Working Group on HEPA Promotion in healthcare settings.

